# Patient-doctor relationship: Changing perspectives and medical litigation

**DOI:** 10.4103/0970-1591.56204

**Published:** 2009

**Authors:** K. Ganesh

**Affiliations:** Directorate General Medical Services (Army), Adjutant General's Branch, Integrated Head Quarters of Ministry of Defence (Army), ‘L’ Block, New Delhi 110 001, India

**Keywords:** Medical negligence, patient-doctor relationship

## Abstract

The patient doctor relational dimer has become complex with the hierarchical or fiduciary manner changing to an equal or un equal relationship. Trust and control are interchangeable, leading to increased patient requirements for disclosure and expectations of a cafeteria approach in diagnoses and management of his/her bodily condition. From any mismatch, there is a potential for medical litigation. In this context, the rise of global consumerism, the explosion of information available on the internet, and the changed manner of the medical profession from being shrouded in mystic / ceremony to trifurcation of medical services to doctoral diagnoses and management, ancillary pharmacy industry, and paramedical services like nursing, counselling and the new age quackery have contributed to this dimer.

The very fact that the article is titled the other way round placing the patient first, even though hoary medical tradition has always demanded that the patient is placed above all else by the treating physician shows that this unusual relationship is in the process of undergoing great transformation. The objective of this article is to see how this patient-doctor dimer is changing and how the changing outlook is contributing to medical negligent claims or litigation.

## ORIGINS OF PATIENT-DOCTOR INTERACTIONS

The origins of the medical profession are steeped in mysticism when doctors more often than not treated their patients free of charge and with contempt. The physician was without reproach and the patient cowered in acceptance of every esoteric or mundane order treating it as God's own gospel.

They functioned from distant temples and patients had to trudge through the countryside to merely access them, often waiting at temple doors for days before the God's own emerged to treat them from their holy precincts. However, with the settled nature of civilisations and the establishment of kingdoms, most Royal houses developed or encouraged their own seers or mendicants who treated the members of the royal family and also allowed their disciples to perfect their art upon the hapless commoners.

From medieval times to the age of the renaissance in modern Europe in the middle of the last millennium, medicine became more codified both in the process of treatment and preparations of cure to the manner in which it was handed over to the patients. One can very well say that the age of democratisation of medical treatment had begun with the doctor now transforming into an elder of society, a guru, but the relationship was fiduciary in nature. Ere the post modern age unto the 19^th^ century the concept of human rights was a piece of poetic imagination. When the polity accepted increasing say of citizenry in political processes, the concept of individual freedom came into acceptance in Western Society. For this to transform itself into patient's rights was not an automatic process, for the nature of the physician-patient relationship being fiduciary and medicine remaining largely in the hands of physicians and their assistants, prevented it.

What contributed to this slow transformation was the age of the Industrial revolution. When modes of communication and transport transformed, the clientele of physicians expanded to include all strata of society. Perforce the load upon physicians increased manifold and resulted in a physician directed division of labor in medical care. The first of these divisions was the founding of the Pharmacological industry, with drug research and development, trial and marketing, and over the counter availability, this took away an important element of physician control over the administration of medication and treatment of clientele. The second development was the advent of nursing, which caught the public's imagination with Florence Nightingale and the ‘lady with the lamp’ was recognised for the emotional and bodily nursing of troops in the Crimean war. With technological advances, the medical sciences accepted laboratory services for pathophysiology and radio-imaging technology. Thus, the medical profession diluted the direct role of the physician and as a result the patient-doctor interface became multifaceted with more and more inputs available to the physician and the patient through the multilayered and nuanced administration of medical care in the post modern era.

## THE SOCIAL TRANSFORMATION OF THE PATIENT-DOCTOR RELATIONSHIP

The social aspects of the doctor-patient relationship have been documented exhaustively beginning with Parsons studies and his formulations. In Western society, Parsons saw four norms governing the functional sick role:

1. The individual is not responsible for their illness
2. Exemption of the sick from normal obligations until they are well
3. Illness is undesirable
4. The ill should seek professional help

This set of formulations was hewn also from the professional mother concept that was common to judiciary and medicine, and hence in early medical litigation the judiciary was deferential to their brother profession of medicine so much as to be prejudiced against patients. Just as the judicial system originated from the one wise man who was pleader, jury, and judge, subsequently through division of labor the Judge came to preside over the pleader and jury in courts of law while retaining pre-eminence, so too the physician automatically retained a pre-eminent perch in the schema for medical care. However, with changing social mores and the free circulation of notes (money) and votes (adult franchise), this social construct was made increasing invalid.

The latest axioms in this field have used Nash's Game theory to create a plausible set of circumstances and responses between the doctor and the patient. The work of Hayes-Bautista (1976) studied bargaining between the patient and the doctor over treatment. The patients were observed using “convincing tactics” of a) demands, b) disclosure that the treatment has not worked, c) suggestions, and d) leading questions. If these did not achieve the desired change in treatment, they turned to “countering tactics” of arguing that the treatment is too weak, too powerful, or insufficient. To augment their authority, the doctors used tactics of a) wielding overwhelming knowledge, b) medical threats about the consequences of ignoring advice, c) disclosures that the treatment may take longer to work for the patient, or d) a personal appeal to the patient as an acquaintance. The outcome measures of this game theoretic situation were a) continuation of the relationship, b) patient termination of the relationship, c) physician termination, and d) mutual termination.

Some authors have gone so far as to predict the premature demise of the patient-doctor relationship by projecting the death of the role of a physician. Maxmen argued that eventually all medical diagnostic and treatment decision-making, the core of the physician's role, would be done better by computers. When this came to be, doctors would no longer be necessary, but rather become a hitherto unknown type of health care professional who would provide the supportive and some of the technical tasks currently being performed by doctors. Because a medic-computer symbiosis would usurp all of the tasks presently assigned to physicians, doctors would be rendered obsolete. This model is a feasible, probable, and desirable alternative future. This scenario is getting a boost with the treatment of Alaskan dental care by the State. In the developing world, the bare foot doctor concept, and in modern armies, the concept of medics is an ongoing program to substitute doctors in certain locations for lesser tasks with technicians. This writer does not subscribe to alarmist American advocates of doctoring the death of the Medical Profession as we know it. Knowledge and training have no substitute as a base for experience to stand on.

So if the doctor will survive the onslaught of the rights activists and itinerant campaigners, then doctors will have to be less guarded about what they are willing to tell their patients, the manner of its telling, the extent of its telling, and who else apart from the patient they must keep in the loop. This is precisely the ground where the new battle lines are drawn.

## LEGAL POSTURE OF LEGITIMATE EXPECTATIONS: FROM BOLAM[1] TO BOLITHO[2] AND BEYOND

Winfield's triad of negligence comprised of 1) the existence of legal duty, 2) breach of legal duty, and 3) damage caused by the breach. Negligence was recognized as a tort only in 1932 with Donohue *vs*. Stevenson.[3] In 1957, in the landmark case of *Bolam vs*. Friern hospital management committee, McNair gave the following directions: “a doctor was liable to be held responsible for negligent act subject to his having acted in a manner that would not be endorsed by a professional body of peers.” In the *Bolitho vs*. City and Hackney Health Authority case, for the first time it was held that peer review alone could not decide medical negligence, but must accommodate patient's expectations in care by allowing rationality in medical decision making, and allowing judicial oversight in medical negligence, with the help of a variety of medical opinion.

The latest judicial trends are pushing the envelope even further, placing greater emphasis upon a patient's right to know and offering patients coherent choices, and the consultation is being looked upon as a business proposition. Recent English case law suggests that the Bolam test is being modified so that a court can reject medical opinion if it is not reasonable or responsible. For example, in Smith *vs*. Tunbridge Wells Health Authority,[4] it was neither reasonable nor responsible for a surgeon not to mention the risk of impotence from rectal surgery, even if some doctors do not mention that risk. The Australian courts have held doctors negligent for failure to disclose risks in a number of cases but a doctor who fails to disclose a material risk will not be held liable on that account alone. Patients must persuade the court that they would not have agreed to the intervention had they been told about the risk, although if a patient is too ill to testify then the jury can substitute the patient's opinion with their own.

Risks should be described in percentage terms where possible, or a broad band or range of figures, rather than by subjective terminology, such as small risk, slight risk, and rare. A risk does not have to be life-threatening to require disclosure. For example, a risk of feces leaking into the vagina, which is unpleasant but not life threatening, must be mentioned. A doctor cannot discharge the duty to inform simply by providing pamphlets about a proposed procedure, such as a pamphlet mentioning capsulation, infection, asymmetry, or change in nipple-breast sensation as risks of breast enlargement.

## HUMANS AS TRAFFIC ISLANDS

The crumbling of traditional societal roles at homes and among the circle of friends and folk, kith and kin saw the collapse of villages as a social unit. Each home has buttressed into a castle and each human as an island. The need for communication has manifested in social networking groups and blogging which is a pretense of communication with no physical social angle or interface. It is still to early to decide what this implosion of communication will have upon society as a whole and humans as individuals. But the trends in the post modern age are that despite the increased modes of communication covering travel, telephony, and telegraphy (web, chatting, email etc.) humans remain prone to loneliness. We are talking more, writing more, and yet appear to be communicating less. This has led to more burdens placed upon professional advisors at all levels, which includes physicians as well. Even if the patient would like to treat the physician with deference, the need for communication overrides all others including that of proper medical care.

## MEDICAL LITIGATION AND THE INDIAN JOURNEY

Though doctors were sued for criminal negligence under the Indian Penal Code, in Dr. Suresh Gupta *vs*. the Govt of NCT of Delhi and others, the Supreme Court,[5] in an appeal on criminal negligence, ruled that mere inadvertence or some degree of want of adequate care and caution might create civil liability but would not suffice to hold the physician criminally liable. Furthermore, the Supreme Court said that for criminal liability there must be a higher degree of negligence or an element of rashness, which could be tantamount to a totally apathetic attitude on the part of the physician towards the patient, for such ‘gross’ negligence alone could the physician be held criminally liable. This rendering of the word “gross” into the IPC Sec 304 A by the Apex Court was decided by a larger bench of the Apex Court in the case of Jacob Mathew *vs*. the State of Punjab and ors,[6] which upheld the decision in Suresh Gupta. With the interpretation of the Consumer Protection Act, 1986, the Supreme Court paved the way for medical litigation in India with the landmark judgement as consumers in VP Shanta *vs*. IMA[7]. The Supreme Court laid down that doctors could be sued for deficiencies in services rendered and defined that all medical consultation fell in the broad swathe of services. Furthermore, the Court went on to describe a distinction between free service and services paid for and said that Consumer Courts could only entertain the latter and not the former. Even in these cases, the Court laid down that any establishment treating even part of its clientele with regular fees was liable to be sued even if the plaintiff had been treated free. However, in a later judgement, the Supreme Court[8] drew a distinction between services rendered free and services rendered by an employer as a contractual obligation as part of conditions of service, by creating medical infrastructure and hosting medical professionals for the purpose and included these establishments under the Consumer Protection Act.

Moreover with the passage of time, Consumer Fora have been emboldened to include all manner of medical litigation and dealt even with negligence as a matter of routine. Furthermore, the Consumer Fora often dispense with the need for expert opinion, and in the interest of limiting the time required to decide cases, do away all together with cross examination of defendants or expert witnesses brought in by medical professionals. The worst is that even though 95% of medical litigation cases are disposed of in favor of the doctor, the compensation for vexatious allegation is almost always never awarded in the context of consumer friendliness. So, like Sita, the medical professional has to emerge unscathed from the fire of these consumer cases time and again, and like her has to fear banishment and needle of suspicion ever after. Reputations take decades to create and running a practice is a life-time investment on part of the medical professional. This can be lost without even an opportunity to be properly examined or allowed legitimate defence. Kerala-based Dr. P.V. George, Past President of the Indian Medical Association questions the concept of a three-tier committee of Consumer Courts that gives verdicts on medical negligence sans knowledge of medicine. To corroborate his stand, he cited a case of a patient in Kerala who succumbed to a cardiac arrest following anesthesia. When the patient's family sued the doctor for medical negligence, the Consumer Court decreed that the patient died of starvation before anesthesia, as advised by the doctor. “The patient could not have died due to starvation prior to anaesthesia, as starvation is a must before anaesthesia” explains Dr George. Medicos vehemently oppose Consumer Courts' practice of accepting cases without any prima facie evidence of negligence. Says New Delhi-based Dr. Vinay Agarwal, secretary general, IMA-national, “Suing a doctor without any prima facie evidence or screening leads to unnecessary travails.” Dr. Agarwal had faced a case of medical negligence in 2003, which was quashed in 2004.

## UROLOGY PRACTICE AND NEGLIGENCE

Examples of some cases that have been successfully claimed against urologists in the course of practice are illustrated below.

1. Negligent performance of anterior colporrhaphy surgery - total loss of vagina. The female plaintiff was 68 years old when she underwent anterior colporrhaphy surgery performed for a cystocoele by the defendant urologist. The National Jury Verdict Review and Analysis (28553) Cook County, Illinois
2. The plaintiff was a 46-year-old male with a long history of impotency who was referred to the defendant urologist for consideration of a penile implant. He also had Peyronie's disease. After the implant, the alleged injury was loss of penis requiring complete reconstruction of penis. The plaintiff claimed record medical compensation. The Virginia Verdict Reporter (126269) Alexandria City
3. A total of 99.9% acetic acid used to treat penile warts caused discoloration. The jury charged under Anderson *vs*. Somberg, the pharmacist, the nurse, the hospital and the urologist. The plaintiff, who was undergoing the removal of penile warts, contended that a 99.9% acetic acid solution, rather than the 4% pH 7.3 diluted solution called for in the procedure, was negligently utilized. The New Jersey Jury Verdict Review and Analysis (19677) Passaic County.
4. W v Barking, Havering, and Redbridge NHS Trust Out of court settlement 17 May 2006. This claim was for negligence in follow-up for successive ureteral stents placed in situ to allow stones to be passed naturally at King George's Hospital and eventually they were removed by ureteroscopy. The encrusted stent could not be removed and required surgery. The Claimant had suffered from incontinence, blood in his urine, and constant pain in his lower back. An expert in Urology concluded that there were repeated and unacceptable delays in providing effective management compounded by questionable clinical decision-making and lack of adequate resources. Departments of Urology have an obligation to maintain a record of patients with uretric stents and ensure that stents are removed or replaced before encrustation and impaction occurs. The Defendants then made an offer of £35,000 which was accepted by the Claimant.

The greatest percent of claims arose from the categories of inpatient, adult, and surgical procedures. Endourological procedures resulted in the greatest incidence of surgical claims. However, claims related to prostatectomy involved the most expensive claims. Of the surgical procedures, incidents defined as postoperative complications were the most common acts of negligence generating a malpractice claim. Urologists must strive to maintain open, honest, in-depth communications with their patients when occurrences with potential malpractice overtones arise.[9]

## THE IPP REPORT

In collaboration, the Commonwealth and the States of Australia appointed a group to review the law of negligence. The Panel was chaired by the Honorable David Ipp, a judge of the Supreme Court of New South Wales. The summaries of recommendations of the Ipp Panel are given below:

- Establishment of thresholds of a percentage of permanent impairment before a person may sue at all.
- Establishment of an indexed maximum for the recovery of economic loss.
- Establishment of a threshold and maximum for recovery of non-economic loss.
- Restrictions on the recovery of damages for gratuitous services.
- Fixing and in all cases reducing the rate of interest that can be awarded.
- Fixing and increasing the discount rate established by the courts for the determination of the present value of future loss.
- Limiting the liability of a volunteer or a Good Samaritan.
- Restricting liability of persons who act in self-defence to criminal conduct.
- Providing that an apology cannot constitute an admission.

Furthermore, the Ipp Panel recommended narrowing duty where breach could be legally examined into: 1) reasonable foreseeability, 2) causation, 3) proportionate liability, and 4) mental trauma.

We need our own ‘Ipp’ committee to review the manner in which our law as well as our courts look at medical negligence and the same is the essence of a lament albeit indirectly in a article written by Dr. Ashok Sinha: “Society is trying to find a solution without assistance from doctors. It was the same when the consumer protection act started. Most of the sane doctors protested, some insane ones also did. No one listened to us. I remember having told one gathering of legal experts that they were putting the patients from the frying pan to fire; from doctors clutches to lawyers. I asked them why they wanted a consumer protection act for the medical community - to improve services or to get compensation, or did they want just to teach a lesson? I assured them none of these would be possible. People refuse to learn from history. Has the road accident compensation policy improved the quality of drivers? It has only raised insurance rates and probably helped the family of the dead. If consumer protection act implementation for medical community was intended for compensation, it was good, but if it was meant to improve services, it was useless.”

## CONCLUSIONS

Thus, it is seen that the march of the tort law defined by generous categorisation of negligence and liability has been limited by the greed for compensation and imagined loss claims that had directly influenced the insurers and the insurance companies by pushing up premiums and increasing the cost of insurance cover to maintain professional practice. It is imperative that the medical practitioners of India also create pressure lobbies to ensure that we don't go the way of Australia or Canada and end up increasing the cost of medical care by way of Mediclaim Premium costs and go full circle only to return to the era of tempered liability vindicated by a Ipp Panel and of modest damages that can be borne by the insurance company and which will be affordable to a medical practitioner by way of premium costs over the length of the medical practitioners' professional lives. For that there is an urgent need to review the functioning of Consumer Fora and their manner of deciding and awarding compensation.

There is an urgent need for medical practitioners to delve into the issue of the patient-physician relationship since this is the bedrock of medical treatment and research. However, since the patient's behavior is causa causens of all medical litigation, as physicians we must offer patients little cause for grief. Let physicians take a little more time in talking, in reassuring patients in the old fashioned way, and restore the patient physician-relationship into one of mutual trust and respect amidst all the disintegration of societal exchanges that are witnessed by the patient. Then the patient will arrive at a new impetus and priority to not disturb any pillar of this patient-physician relationship. That would be ideal, and that would be a responsible way of looking at medical litigation. If this calls for a rejig of our medical training, medical care, and delivery system then so be it. Let it be treated as the Clarion call, ‘PHYSICIAN HEAL THYSELF’!
